# Supplementary material for: Effectiveness of interactive teaching intervention on medical students’ knowledge and attitudes toward stem cells, their therapeutic uses, and potential research applications
Source: PeerJ. 2022 Jan 21;10:e12824. doi: 10.7717/peerj.12824 (PMC8785657; doi:10.7717/peerj.12824)
Supplement: Supplemental Information 1 [file peerj-10-12824-s001.pdf]

If you are a sixth-year medical student at the University of Science and Technology Yemen-Jordan branch (USTY-Jo) and would like to participate in this study, please fill this questionnaire. Your accurate answers will be appreciated.

Participant ID code (Please provide it exactly as written in the consent form and previous filled survey):

………………………………………………………………

** Remember: The participant ID code should consist of the first letters of your first and family names (A–Z) plus a four-digit code that represents birthday (01-31) and month of birth (01-12).*

Section 1: Respondents' Demographic Characteristics.

**Age:**				
**Gender:**		Male		Female
**Nationality:**				
**Name of your medical school:**				
**Year Level:**				

Section 2: Students' Knowledge about Stem Cells.

How would you rate your knowledge regarding stem cells in general?

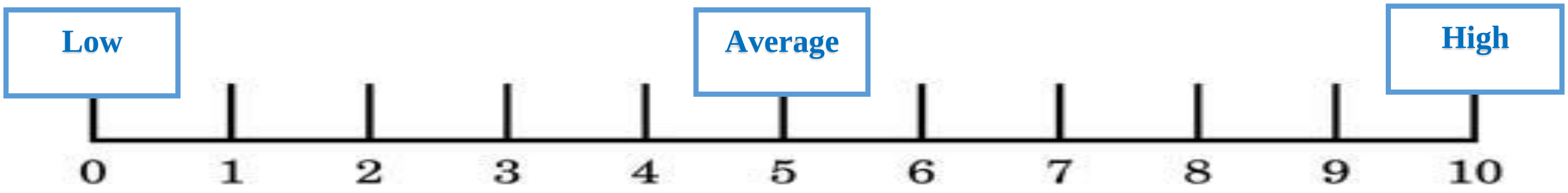

Your knowledge regarding stem cells is derived from which of the following sources (choose all that apply):

	Books		Medical journals		Workshops		Social Media
	Lectures		Medical conferences		Panel discussions		Other sources
	Please mention the other sources for your knowledge about stem cells:…………………						

The following statements assess students' knowledge regarding stem cells, their potential applications, therapeutic uses, and research involving them. These statements consist of eleven correctly stated statements, seven false or misleading statements, and neither true nor false statements, with a total of nine statements.

Statements: How much do you agree or disagree with these statements?	Strongly Agree (SA)	Agree (A)	Neutral (N)	Disagree (D)	Strongly Disagree (SD)
**Stem Cells: Basic Knowledge**					
1- I have sufficient knowledge of different types of stem cells, such as adult and embryonic stem cells.	**SA**	**A**	**N**	**D**	**SD**
2- I have sufficient knowledge of sources of stem cells.	**SA**	**A**	**N**	**D**	**SD**
3- I have sufficient knowledge of the therapeutic uses of stem cells.	**SA**	**A**	**N**	**D**	**SD**
4- I have sufficient knowledge of the three germ layers (endoderm, mesoderm, and ectoderm) and organs and tissues generated from each layer.	**SA**	**A**	**N**	**D**	**SD**
5- Cell differentiation is the process by which stem cells become more specialized cell types.	**SA**	**A**	**N**	**D**	**SD**
6- As a stem cell differentiates, it gradually loses potency and becomes unipotent.	**SA**	**A**	**N**	**D**	**SD**
7- Self-renewing is the ability of a stem cell to produce more stem cells with identical characteristics as the "parent" cell.	**SA**	**A**	**N**	**D**	**SD**
8- Adult stem cells are pluripotent cells that have the potential to make all cell types of the body.	**SA**	**A**	**N**	**D**	**SD**
9- Bone marrow is the only source for adult stem cells.	**SA**	**A**	**N**	**D**	**SD**
10- Stem cells can differentiate into many cell types within a germ layer.	**SA**	**A**	**N**	**D**	**SD**
11- Embryonic stem cells are derived from leftover blastocysts after in vitro fertilization.	**SA**	**A**	**N**	**D**	**SD**
12- Embryonic stem cells are derived from the umbilical cord after childbirth.	**SA**	**A**	**N**	**D**	**SD**
13- Embryonic stem cells are derived from the trophoblast of blastocysts.	**SA**	**A**	**N**	**D**	**SD**

Statements: How much do you agree or disagree with these statements?	Strongly Agree (SA)	Agree (A)	Neutral (N)	Disagree (D)	Strongly Disagree (SD)
**Stem Cells: Potential Applications**					
14- Stem cells can be used to study early human development.	SA	A	N	D	SD
15- Stem cells can be used to understand the pathophysiology and analyze disease mechanisms by modeling disease in a culture dish outside the human body.	SA	A	N	D	SD
16- Stem cells can be used to test and screen new drug candidates and toxins to figure out their potential side effects.	SA	A	N	D	SD
17- Stem cells can be used to replace or restore tissues that have been damaged by disease or injury, such as diabetes, heart attacks, Parkinson's disease, skin burns, or spinal cord injury.	SA	A	N	D	SD
**Stem Cells: Therapeutic Uses**					
18- There is a wide range of conditions or diseases for which stem cell therapies have been proven to be safe and effective such as osteoarthritis and multiple sclerosis.	SA	A	N	D	SD
19- There is nothing to lose from trying unproven stem cell therapies since they can provide hope for hopeful patients.	SA	A	N	D	SD
20- Bone marrow-derived stem cells will spontaneously regenerate into different cell types such as hepatocytes and neural cells without manipulation in the lab.	SA	A	N	D	SD
21- If the balance is skewed between differentiation and self-renewing properties of stem cells, it may result in tumor formation.	SA	A	N	D	SD
**Stem Cells: Research**					
22- I would be confident to explain the induced-Pluripotent Stem Cells (iPSCs).	SA	A	N	D	SD
23- I would be confident to explain the transcription factors.	SA	A	N	D	SD
24- Adult cells can be “reprogrammed” genetically to assume a stem cell-like state.	SA	A	N	D	SD
25- I would be confident to discuss the Somatic Cell Nuclear Transfer (SCNT).	SA	A	N	D	SD
26- I would be confident to explain the differences between therapeutic cloning and reproductive cloning.	SA	A	N	D	SD
27- I would be confident to discuss mitochondrial replacement therapy.	SA	A	N	D	SD

Section 3: Students' Attitudes Regarding Stem Cells.

The following statements assess students' attitudes and perceptions regarding stem cells.

**Statements: How much do you agree or disagree with these statements?**	**Strongly Agree (SA)**	**Agree (A)**	**Neutral (N)**	**Disagree (D)**	**Strongly Disagree (SD)**
1- I am interested in expanding my knowledge about stem cells.	**SA**	**A**	**N**	**D**	**SD**
2- Stem cell education should be integrated into medical college curricula.	**SA**	**A**	**N**	**D**	**SD**
3- I would consider a well-structured program or training focusing on stem cell science.	**SA**	**A**	**N**	**D**	**SD**
4- I think stem cell therapies give rise to ethical controversies.	**SA**	**A**	**N**	**D**	**SD**
5- I think stem cell therapies give rise to religious controversies.	**SA**	**A**	**N**	**D**	**SD**
6- Government should spend money to support stem cell research.	**SA**	**A**	**N**	**D**	**SD**
7- Transitional process of taking stem cell therapy from the laboratory through clinical trials should be encouraged.	**SA**	**A**	**N**	**D**	**SD**
8- People should consider the donation of bone marrow to a public bank.	**SA**	**A**	**N**	**D**	**SD**
9- People should consider the donation of their babies' umbilical cord blood for a public bank.	**SA**	**A**	**N**	**D**	**SD**
10- I am willing to pay money for preserving the umbilical cord blood of my baby in a private bank for later use if a therapeutic need arises.	**SA**	**A**	**N**	**D**	**SD**

Thank you for your time. Please write any comments below:
